# Associations between Systemic Sclerosis and Thyroid Diseases

**DOI:** 10.3389/fendo.2017.00266

**Published:** 2017-10-03

**Authors:** Poupak Fallahi, Ilaria Ruffilli, Dilia Giuggioli, Michele Colaci, Silvia Martina Ferrari, Alessandro Antonelli, Clodoveo Ferri

**Affiliations:** ^1^Department of Clinical and Experimental Medicine, University of Pisa, Pisa, Italy; ^2^Department of Medical, Surgical, Maternal, Pediatric and Adult Sciences, University of Modena and Reggio Emilia, Modena, Italy

**Keywords:** systemic sclerosis, autoimmune thyroiditis, hypothyroidism, Graves’ disease, thyroid cancer, antithyroperoxidase antibodies, antithyroglobulin antibodies, CXCL10

## Abstract

We have reviewed scientific literature about the association of systemic sclerosis (SSc) and thyroid disorders. A high incidence, and prevalence, of new cases of autoimmune thyroiditis (AT) and/or hypothyroidism have been shown in sclerodermic patients (overall in the female gender). An association among a Th1 immune-predominance, low vitamin D levels, and AT have been also shown in SSc patients. Cases of Graves’ disease (GD) have been described in SSc patients, too, according with the higher prevalence of thyroid autoimmunity. It has been also shown a higher prevalence of papillary thyroid cancer (PTC), in association with AT, in SSc patients. However, in order to confirm results about GD and thyroid cancer, studies in larger number of patients with SSc are needed. During the follow-up of SSc patients it would be appropriate to monitor carefully their thyroid status. The abovementioned data strongly suggest a periodic thyroid function follow-up in female SSc patients [showing a borderline high (although in the normal range) thyroid-stimulating hormone level, antithyroid peroxidase antibody positivity, and a small thyroid with a hypoechoic pattern], and, when necessary, appropriate treatments. In conclusion, most of the studies show an association among SSc, AT, and hypothyroidism, such as an increased prevalence of TC overall in SSc patients with AT. Only few cases of GD have been also described in SSc.

## Introduction

Systemic sclerosis (SSc) is a connective tissue disease characterized by degenerative microvascular phenomena and immune system activation, that lead to fibrosis of the skin and internal organs (1, 2). SSc is clinically a multifaceted disorder derived from different contributions of the abovementioned pathogenetic mechanisms, through a multistep process that causes various clinical phenotypes (3). SSc is a heterogeneous autoimmune disease which has defined by three hallmarks: small vessel vasculopathy, production of autoantibodies, and fibroblast dysfunction. The exact etiology of the disease remains unknown, due to the complex nature of the cellular signaling pathways involved. However, there is strong and consistent evidence that the innate system, in particular toll-like receptor signaling, is contributing to the progression and perhaps onset of SSc (4).

Two overlapping SSc forms exist: (a) a limited cutaneous scleroderma (lSSc), confined to the skin of face, hands and feet and (b) a diffuse cutaneous scleroderma (dcSSc), extended over other areas of the skin, that can involve visceral organs, as kidneys, lungs, heart, and gastrointestinal tract. Patients affected by the limited form show a good prognosis, with a 10-year survival in about 75% of patients; however, about 10% of them develop pulmonary arterial hypertension after 15 years. Patients with dcSSc have a 10-year survival of 55%; death is commonly associated with pulmonary, heart, and kidney involvement.

The diagnosis is established on the basis of clinical suspicion, the presence of autoantibodies (in particular anticentromere and anti-scl70/antitopoisomerase antibodies) and quite rarely on biopsy. Regarding the antibodies, 90% of SSc patients have a detectable antinuclear antibody; anticentromere antibody is more frequent in lSSc (80–90%) compared to dcSSc (10%), and anti-scl70 is more common in dcSSc (30–40%) (5).

The American College of Rheumatology set the diagnostic criteria for scleroderma in 1980 (6).

Systemic sclerosis is associated with significant morbidity (including skin thickening, finger ulcers, joint contractures, pulmonary fibrosis and hypertension, chronic diarrhea, and renal failure) (7).

Systemic sclerosis patients have high rates of symptoms of depression, and SSc is associated with substantially reduced health-related quality of life (8).

Many studies show a high prevalence of autoimmune thyroid disorders (AITDs) in SSc patients; however, contradictory results have been reported too. Here, we review the scientific literature about the possible association of SSc with autoimmune thyroiditis (AT), Graves’ disease (GD), and also thyroid cancer (TC).

## SSc and AT

After the initial case reports (9, 10), the association of SSc and AITD has been evaluated by many studies (Table 1).

**Table 1:** Prevalence of thyroid autoimmunity in SSc patients versus controls, in the published studies that included an internal control group.

Reference	SSc patients (*n*)	AITD% in SSc patients	Controls (*n*)	AITD% in controls	*P*
Gordon et al. (11)	56	14	56	2	<0.05
Shahin et al. (17)	24	Serum levels of FT4 in patients were significantly lower than in controls (7.46 ± 2.7 for patients versus 10.5 ± 1.8 for controls with *P* < 0.001). Of the 24 patients, 8 (33.3%) showed hypothyroidism, evidenced by decreased FT4 and increased TSH beyond normal ranges, 5/14 (32.7%) with dSSc versus 3/10 (30%) with lSSc (*P* = ns)	15		
Innocencio et al. (18)	25	52	113	3.5	
Antonelli et al. (20)	F 184; M 18	F 107/77 (58%); M 7/11 (39%)	F 368; M 36	F 100/268 (27%); M 2/34 (6%)	F 0.0001; M 0.0019
Marasini et al. (21)	79	AbTg in 14% of patients; AbTPO in 23% of patients	81 women with OA serving as controls	AbTg in 13% of patients; AbTPO in 11% of patients	ns (for AbTg); 0.057 (for AbTPO)
Danielides et al. (23)	138	AbTPO and/or AbTg positive in 29% of patients	100	AbTPO and/or AbTg positive in 29% of controls	ns
Antonelli et al. (25)	179	24% (initial thyroid status); 34% (last thyroid status)	179	12% (initial thyroid status); 16% (last thyroid status)	0.002 (initial thyroid status); <0.001 (last thyroid status)

*AbTg, antithyroglobulin antibodies; AbTPO, antithyroperoxidase antibodies; F, female; FT4, free thyroxine; M, male; ns, not significant; OA, osteoarthritis; TSH, thyroid-stimulating hormone*.

A first systematic study (11) reviewed patients with fatal SSc about pathologic and serologic evidence of thyroid disorders. Histologic evidence of severe fibrosis of the thyroid was reported in 14% of 56 SSc cases (versus 2% of age- and gender-matched control autopsy series). Among 27 SSc patients in whom thyroid-stimulating hormone (TSH) and free thyroid hormones were measured, 7 (26%) were hypothyroid, and 9 had euthyroid sick syndrome. Hypothyroid patients had thyroid glands with fibrosis, but a few lymphocytic infiltration. However, 6/7 of hypothyroid patients had elevated levels of circulating antithyroglobulin antibodies (AbTg). These findings suggested a thyroid autoimmune process leading to gland fibrosis and hypothyroidism in severe SSc patients.

A second study (12) found decreased free thyroxine (FT4), decreased free triiodothyronine (FT3), and increased TSH in 42 SSc patients, versus age and gender controls. However, changes in FT4, FT3, and TSH were small with mean values within normal ranges, suggesting a subclinical thyroid dysfunction.

In a further study, 77 SSc patients were evaluated by measurements of basal FT4, FT3, TSH, and the TSH response to thyrotropin-releasing hormone (13). Eight patients (10%) were hypothyroid. Antithyroid antibodies (ATA) were present in four of eight (50%) of the hypothyroid patients.

Among 39 SSc patients (14), 2 patients had clinical hypothyroidism, while 7 had subclinical hypothyroidism. On the whole 9/39 (23%) of SSc patients were hypothyroid, and 4/9 (44%) had positive ATA. Circulating AbTg and/or antimicrosomal antibodies were positive in 18% of the 39 patients.

A high prevalence of thyroid autoimmunity (15) was also observed in 43 Hungarian SSc patients. ATA were detected in 14 cases (33%) [4 cases with AbTg, 11 with antithyroperoxidase antibodies (AbTPO), and 5 with antimicrosomal antibodies].

Antithyroglobulin antibodies and AbTPO antibodies, FT3, FT4 and TSH, and HLA-DR typing were carried out in 85 SSc Italian patients (16). AbTg and AbTPO antibodies were detected in 12% (10/85) and 19% (16/85) of patients. Two patients with ATA had clinical hypothyroidism and shared the HLA-DR3 allele. A higher frequency of the HLA-DR15 was shown in SSc subjects in the presence of AbTPO antibodies than in patients without AbTPO.

Twenty-three female patients with SSc (mean age 37.7 ± 12.7) were evaluated for thyroid dysfunctions versus 15 normal females as controls (17). Mean serum levels of FT4 in SSc patients were significantly lower than in controls (7.46 ± 2.7 versus 10.47 ± 2.5). Of the 24 patients, 8 (33.3%) showed hypothyroidism (17).

Another Latin American study confirmed a high prevalence of thyroid autoantibodies in Brasilian patients with SSc (18).

In a large cohort study (19), 1,517 patients with rheumatoid arthritis (RA), systemic lupus erythematosus (SLE), SSc, Sjögren’s syndrome (SS), mixed connective tissue disease (MCTD), and polymyositis/dermatomyositis (PM/DM) were evaluated for thyroid dysfunctions clinically and by imaging and fine-needle aspiration cytology [with respect to prevalence of GD or Hashimoto’s thyroiditis (HT) in the general population]. HT was more common among MCTD, SS, and RA patients (21, 7, and 6%, respectively) than GD (2.5, 3, and 1.6%, respectively). SLE, RA, SSc, MCTD, SS, and PM/DM had a higher prevalence for HT than the general population of 90-, 160-, 220-, 556-, 176-, and 69-fold, respectively, and for GD of 68-, 50-, 102-, 76-, 74-, and 37-fold, respectively.

A first study (20) aimed to assess the prevalence of thyroid disorders in SSc patients using a complete thyroid work-up, versus an internal appropriate control group. Two hundred two SSc patients versus 404 controls from the general population (matched by age and gender, with similar iodine intake) were evaluated for TSH, FT3, FT4, AbTg and AbTPO, thyroid ultrasonography and blood flow, and fine needle aspiration when needed. Odds ratios (OR) for female SSc versus controls subjects were significant for: clinical hypothyroidism, 14.5 (2.3–90.9); subclinical hypothyroidism, 3.2 (1.8–5.7); AbTPO positivity, 2.7 (1.8–4.1); thyroid hypoechoic pattern, 3.2 (2.2–4.7); thyroid autoimmunity, 3.7 (2.6–5.4); and thyroid volume <6 mL, 1.8 (1.2–2.7). OR for thyroid autoimmunity in male SSc patients versus control subjects was 10.8 (2.2–52.4). Female SSc patients had mean TSH levels significantly higher than control subjects, and female and male SSc patients had significantly higher AbTPO than controls. Three cases of GD in female SSc (versus zero in controls, *P* < 0.05), and two of papillary thyroid cancer (PTC) were reported in SSc patients.

The authors suggested to test thyroid function, AbTPO, and ultrasonography and to assess the clinical profile of SSc patients. Thyroid function follow-up (and appropriate treatments) should be performed periodically in women, subjects with positive AbTPO and hypoechoic and small thyroid.

The abovementioned results were confirmed in another study (21) that evaluated thyroid function and autoantibodies, in 79 SSc women, versus 81 age-matched women with osteoarthritis (OA) as controls. Hypothyroidism was found in 16 of 79 (20%) patients with SSc and in 9 of 81 (11%) patients with OA. AbTPO were present in 23% SSc versus 11% controls. The risk of hypothyroidism was significantly higher in AbTPO-positive patients (*P* < 0.0001).

A cross-sectional study (22) of two convenience samples of patients with SSc, one in Canada and the other in Colombia, was performed. Among 719 patients, 273 (38%) had at least one other autoimmune disease. Three hundred sixty-six autoimmune diseases were assessed, among which AITD (38%), RA (21%), SS (18%), and primary biliary cirrhosis (4%) were more frequent. Two hundred sixty patients (36%) had first-degree relatives with at least one autoimmune disease [RA (18%) and AITD (9%) were the most common]. These results suggest that polyautoimmunity is frequent in SSc patients and autoimmune diseases cluster within families of these patients.

To determine (23) the ATA prevalence in a large SSc cohort and to verify whether they are associated with distinct clinical phenotypes, 138 SSc patients (46 with dSSc and 92 with lSSc) and 100 healthy controls (HC) were tested for AbTg and AbTPO. A statistically significant increase of AbTPO was detected only in patients with lSSc compared to HC (32.6 versus 14%, *P* = 0.003).

Two hundred ten SSc patients were evaluated in a Japanese study (24), that identified 30 patients with AITD (14.3%), including 29 with HT (13.8%) and 1 with GD (0.5%).

A further study (25) first evaluated the incidence of new cases of clinical and subclinical thyroid dysfunction in SSc women versus controls from the same geographic area (matched by gender and age). SSc patients with thyroid dysfunction were excluded at the beginning, and then the manifestation of new cases of thyroid disorders was assessed in 179 patients and 179 matched controls, having a similar iodine intake (median follow-up: 94 months in controls; 73 months in SSc). An elevated incidence (*P* < 0.05) of new cases of hypothyroidism, thyroid dysfunction, AbTPO positivity, and hypoechoic thyroid, in SSc patients (15.5, 21, 11, and 14.6 of 1,000 patients per year; respectively) versus those in controls was observed. The onset of hypothyroidism was demonstrated (by logistic regression analysis) to be related to a borderline elevated initial TSH value, the presence of high AbTPO levels, and a hypoechoic and small thyroid in SSc patients.

A subsequent study (26) confirmed a high prevalence of subclinical hypothyroidism (8.5%), overt hypothyroidism (1.9%), subclinical hyperthyroidism (2.8%), and overt hyperthyroidism (0.9%) in SSc patients, and that a small thyroid volume (<4.5 ml) was related to hypothyroidism.

Positive ATA titers were also observed in 27/86 SSc Polish (27) patients (31%).

Recently, it has been also demonstrated that hypovitaminosis D was statistically associated with AT in SSc patients (28).

Conversely, a recent study (29) evaluated prospectively the prevalence of other autoimmune disorders in outpatient clinic in 3,069 consecutive patients with diagnosed chronic AT, with respect to two control groups (matched by age and gender): (a) a control group of 1,023 subjects, drawn out a random sample of the general population without thyroid disorders and (b) 1,023 patients with non-toxic multinodular goiter extracted from the same random sample of the general population, with similar iodine intake. The results of the study demonstrated a significant increase of the prevalence of SSc in AT patients (with respect to both controls).

Different studies demonstrate elevated circulating CXCL10 (Th1) and CCL2 (Th2) chemokines in SSc patients of newly diagnosis. Patients with a serious clinical phenotype, including the involvement of lung and kidney, have higher CXCL10. CXCL10 declines during the follow-up, while CCL2 does not change, suggesting the progress from a beginning Th1 inflammatory stage to a successive Th2 phase (30, 31).

Th1 lymphocytes, interferon-γ, and interferon-γ-dependent chemokines (CXCL9, CXCL10, CXCL11) play a pivotal role in AITD, that are Th1 immune-mediated autoimmune disorders, too (32–35). Newly diagnosed SSc patients have elevated circulating levels of CXCL10, but not of CCL2, in the presence of AT, indicating a predominance of the Th1 immune response in these patients (36).

To sum up, the abovementioned data show a high incidence, and prevalence, of new cases of AT, and hypothyroidism, in patients with SSc, suggesting that in SSc women, with a borderline high (though in the normal range) TSH level, in the presence of AbTPO, and a hypoechoic and small thyroid, it could be necessary to monitor periodically thyroid function.

## SSc and GD

A first anectodal study reported an association of SSc and GD in three cases (37). One case of GD was also observed among 210 SSc Japanese patients (0.5%) (24). Graves’ ophthalmopathy has been also occasionally reported in one SSc patient (38).

A significant number (3 cases) of GD in female SSc (3/202 versus 0/404 controls, *P* < 0.05) was also observed in a case control study (20), with an internal appropriate control group.

On the whole the abovementioned studies suggest a higher prevalence of GD in SSc patients; nevertheless further studies, involving a larger number of SSc patients, are necessary to confirm this finding.

## SSc and PTC

Single cases of PTC in association with SSc were reported in several studies (9, 20, 39–42).

However, more recently, the risk of TC in 327 unselected SSc patients with respect to two population-based, control groups was studied systematically (matched by age and gender; 654 subjects from an iodine-deficient area and 654 subjects from an iodine-sufficient area) (43).

Six subjects with PTC were detected among SSc patients, while only one case was observed in controls 1, as well in controls 2 (*P* = 0.007, for both). In SSc, all patients with TC showed thyroid autoimmunity versus 40% of the other SSc patients (*P* = 0.001) (43).

These findings suggest the possibility of a increased prevalence of PTC in SSc patients with thyroid autoimmunity, however, larger cohorts are needed to elucidate this.

## Clinical Aspects of SSc and Thyroid Disorders

Several studies have evaluated a possible association among thyroid disorders and clinical findings of SSc, reporting different results.

It was initially reported that patients with hypothyroidism had more frequently subcutaneous calcinosis (11).

Hungarian SSc patients with AbTPO concentration tended to have secondary SS (15).

In a further study in female patients with SSc (duration < 3 years), FT4 levels correlated significantly with Dlco% (*r* = + 0.90, *P* < 0.01), while in patients with SSc duration > 3 years hypothyroidism correlated significantly with hand joint restriction of motion (17).

Seventeen SSc patients with high pulmonary systolic pressure (>35 mmHg) were studied in another article (44). High pulmonary pressure in these SSc patients was not associated with the type of SSc, or age; however, five SSc patients (12.5%) had alteration of the thyroid function (two cases of hypothyroidism, three of hyperthyroidism). The pulmonary pressure levels were higher in SSc patients with thyroid dysfunction, with respect to SSc in euthyroidism (40 versus 31 mmHg, respectively; *P* < 0.05). Furthermore, hypothyroid SSc patients had higher pressure levels, with respect to hyperthyroid SSc (46 versus 37 mmHg, respectively), even if not significantly. The lack of significant differences in pressure levels between hypothyroid, or hyperthyroid SSc, and the low frequency of ATA in pulmonary hypertension associated with SSc, suggest a vasomotor role of thyroid hormones, rather than an autoimmune mechanism (44).

In Japanese female SSc patients, the prevalence of anticentromere antibody positivity, SS, and severe facial skin sclerosis was significantly prevalent in the presence of AITD (24).

A study found a statistically significant increase of AbTPO only in patients with lSSc (but not in dSSc) compared to controls (32.6 versus 14%, *P* = 0.003) (23).

To sum up, most of the abovementioned studies did not observed any association among thyroid dysfunctions and/or autoimmunity and features of SSc (clinical or serological) (13, 20, 25, 27).

## Conclusion

Many studies have shown a high incidence, and/or prevalence, of new cases of AT, and hypothyroidism in SSc patients, especially in the female gender. An association among a Th1 predominance, low levels of vitamin D, and AITDs has been also demonstrated in patients with SSc.

Few cases of GD have been also described in SSc patients, according with the higher prevalence of thyroid autoimmunity.

It has been also observed a higher prevalence of PTC, in association with AT, in SSc patients. However, in order to confirm results about GD and TC, studies in larger number of patients with SSc are needed.

To sum up, the abovementioned data strongly suggest that female SSc patients with a high risk (a borderline high though normal TSH value, the presence of AbTPO, and a hypoechoic and small thyroid) should be periodically monitored for thyroid function, and appropriate treatments when needed.

## Author Contributions

PF, IR, DG, MC, SMF, AA, and CF gave substantial contribution in the conception and design of the work, and in writing the article. AA and CF revised it critically for important intellectual content. PF, IR, DG, MC, SMF, AA, and CF gave the final approval of the version to be published. PF, IR, DG, MC, SMF, AA, and CF agreed to be accountable for all aspects of the work in ensuring that questions related to the accuracy or integrity of any part of the work are appropriately investigated and resolved.

## Conflict of Interest Statement

The authors declare that the research was conducted in the absence of any commercial or financial relationships that could be construed as a potential conflict of interest.

## Abbreviations

AbTg, antithyroglobulin antibodies; AbTPO, antithyroid peroxidase antibodies; AITD, autoimmune thyroid disorders; AT, autoimmune thyroiditis; ATA, antithyroid antibodies; dcSSC, diffuse cutaneous scleroderma; FT3, free triiodothyronine; FT4, free thyroxine; GD, Graves’ disease; HC, healthy controls; HT, Hashimoto’s thyroiditis; lSSc, limited cutaneous scleroderma; MCTD, mixed connective tissue disease; OA, osteoarthritis; OR, odds ratios; PM/DM, polymyositis/dermatomyositis; PTC, papillary thyroid cancer; RA, rheumatoid arthritis; SLE, systemic lupus erythematosus; SS, Sjögren’s syndrome; SSc, systemic sclerosis; TC, thyroid cancer; TSH, thyroid-stimulating hormone.
